# Enhancing cotton sustainability: Multi-factorial intercropping, irrigation, and weed effects on productivity, quality and physiology

**DOI:** 10.1016/j.heliyon.2024.e27135

**Published:** 2024-02-27

**Authors:** Basim Mohammed Abdulkareem, Ali Mokhtassi-Bidgoli, Mahdi Ayyari, Eshagh Keshtkar, Hamed Eyni-Nargeseh

**Affiliations:** a Department of Agronomy, Faculty of Agriculture, Tarbiat Modares University, PO Box 14115-336, Tehran, Iran; b Department of Horticultural Science, Tarbiat Modares University, Tehran, Iran; c Department of Agricultural Science, Technical and Vocational University (TVU), Tehran, Iran

**Keywords:** Fiber plant, Medicinal plant, Replacement series, Nutritional composition, Weed suppression

## Abstract

Drought stress and weed infestation are significant factors that significantly decrease cotton yield. Increasing the variety of plants within a cotton field ecosystem can strengthen its stability and protect it from susceptibility to both biotic and abiotic pressures. In this two-year experiment (2021 and 2022), the effects of intercropping systems (four growth conditions including mono- and inter-cropped cotton varieties Golestan and Hekmat with *Nepeta crispa* and dragon's head (*Lallemantia iberica*)), irrigation (three intervals of 3, 6, and 9 days), and weed competition (weed-free and weedy plots) on the agronomic performance, physiological characteristics, and seed quality of cotton in a semi-arid region of Iran were studied. In 2021, the volume of irrigation water applied was 9873, 6100, and 4650 m^3^ ha^−1^ for irrigation intervals of 3, 6, and 9 days, respectively. In 2022, the volumes were 9071, 5605, and 4272 m^3^ ha^−1^ for the corresponding irrigation intervals. Over two years, *Xanthium strumarium*, *Amaranthus retroflexus*, and *Portulaca oleracea* were the dominant weed species. Weeds had the most significant impact on total dry weight; weed control increased plant vigor and growth, ranging from 1.4 to 2.3 times, while weed impact on cottonseed yield ranged from 18% to 96% reduction. Increasing irrigation intervals resulted in reductions in various parameters, with decreases of 39%–80% in total dry weight, 34%–57% in cottonseed yield, and 48%–72% in lint yield. The harvest indices for seed cotton, cottonseed, and lint ranged from 35.3% to 56.5%, 18.3%–35.0%, and 15.4%–20.5%, respectively. Weeds were responsible for a 17% decrease in the 1000-seed weight. As the irrigation intervals increased from 3 days to 6 days and 9 days, the number of bolls per plant decreased by 19%–85%. Extending the irrigation interval from 3 days to 6 days and 9 days resulted in a substantial decrease in the photosynthetic rate, ranging from 42% to 92%. Mono-cropped Golestan performed well under unstressed conditions such as 3-day interval irrigation and weed-free conditions. On the other hand, intercropped Hekmat demonstrated better resilience to both moisture and weed stresses. The LER (Land equivalent ratio) indices of both intercropping systems were generally favorable, indicating higher productivity compared to sole cropping. The intercropping systems consistently showed the highest LER indices under weedy conditions, highlighting the significance of intercropping as a valuable method in integrated weed management.

## Highlights

• *Xanthium strumarium* decreased 13%, while *Portulaca oleracea* and *Setaria viridis* showed 7% and 10% increases in warmer, drier year.
• Weed control increased plant vigor and growth, ranging from 1.4 to 2.3 times.
• Intercropping systems consistently produced taller plants, but weed presence and longer irrigation intervals reduced plant height.
• Extending irrigation intervals from 3d to 6d and 9d resulted in substantial reductions in photosynthetic rate, ranging from 42% to 92%.
• The intercropping systems consistently showed the highest land equivalent ratios under weedy conditions.

## 1 Introduction

Designing planting patterns and incorporating intercropping practices are crucial activities for enhancing the sustainability of agricultural ecosystems. Intercropping refers to planting a second crop alongside the primary crop within agricultural systems [1]. By adopting intercropping methods, farmers can optimize resource use, promote biodiversity, reduce reliance on synthetic inputs, and mitigate environmental degradation. Embracing these practices not only ensures long-term food security, but also supports the preservation of our natural resources for future generations [2,3]. Many medicinal plants are threatened by habitat loss and overexploitation. By incorporating them into intercropping systems, farmers can help preserve these plants while benefiting from their ecological and economic advantages. Two notable medicinal plants from the Lamiaceae family are *Nepeta crispa* and dragon's head (*Lallemantia iberica* (M. Bieb.) Fisch. & C.A. Mey). These plants are highly regarded for their significant contributions to medicinal products and promoting community health [4,5].

Cotton is a major cash crop with a significant role in global trade and generates substantial revenue in China, India, the United States, Pakistan, Brazil, Australia, Uzbekistan, Turkey, and Africa [[6], [7], [8]]. According to the Food and Agriculture Organization (FAO), the world's total seed cotton production was around 83 million tons in 2020. Overall, cotton's significance and contributions extend across multiple sectors and industries like textile, oil, food, and livestock, making it an essential and highly valued resource [9].

Increasing the variety of plants can strengthen ecosystem stability and protect it from being susceptible to both living (biotic) and non-living (abiotic) pressures [10]. Drought stress and weed infestation are significant factors in both abiotic and biotic stresses [[11], [12], [13], [14]]. These can significantly decrease cotton yield [15]. The extent of the impact depends on factors such as the severity and duration of drought, the type and competitiveness of weeds, and the stage of cotton growth when these factors occur [16,17]. Furthermore, weeds can use 30–50% of applied fertilizer and can also reduce moisture levels by 20–40% [6]. In the USA, weeds pose a significant challenge in pest management, with 90% of USA farmers and consultants identifying weed management as a top three constraint. The most common weeds in organic cotton fields in the USA, listed in order of prevalence, include bindweed (*Convolvulus arvensis* L.), pigweed (*Amaranthus* spp.), lakeweed (*Polygonum hydropiper* L.), johnsongrass (*Sorghum halepense* (L.) Pers.), morningglory (*Ipomoea purpurea* (L.) Roth), nut grass (*Cyperus rotundus* L.), and crabgrass (*Digitaria* spp.) [18]. For example, cotton crops have experienced significant yield reductions of between 6 and 65% due to the presence of Palmer amaranth (*Amaranthus palmeri*) throughout the growing season in many cotton production areas in the USA [19]. In an experiment conducted by Hakoomat et al. [9], implementation of manual weeding led to a 2.4-fold increase in seed cotton yield versus the control treatment. Additionally, certain weeds in cotton are displaying resistance to herbicides [20]. Moffett and McCloskey [16] conducted a three-year study where they discovered a reduction in irrigation water 55% (from 166 cm to 107 cm) led to a decrease in seed cotton yield of 20% (from 4005 kg/ha to 3324 kg/ha). Irrigated cotton in Australia consistently achieved an average yield that was 2.8 times higher than raingrown cotton, highlighting the significant impact of water stress on crop productivity [7].

Effective irrigation practices, adoption of drought-tolerant cotton varieties, and integrated weed management approaches, including adhering to plant quarantine regulations, implementing cultural and managerial techniques, employing both biological and chemical approaches, and utilizing manual and mechanical weed control, can help mitigate these negative impacts and improve cotton production under challenging conditions [20]. In this regard, the cumulative benefits of choosing a stress-tolerant and competitive cultivar will be evident through various positive outcomes, including a significant reduction in environmental pollution, lower production costs, and the promotion of sustainable food production [15]. Intensive cultivation of the crops expressed in intercropping systems achieved lower weed infestation while higher yield potential than sole cropping [[21], [22], [23]]. Rajpoot et al. [24] reported that weed biomass experienced significant reductions of approximately 57% and 265% in intercropping systems involving cotton + okra (*Abelmoschus esculentus* (L.) Moench) and cotton + cowpea (*Vigna unguiculata* (L.) Walp.), respectively, when compared to the cultivation of sole cotton. It was found that intercropping sesame (*Sesamum indicum* L.), soybean (*Glycine max* [L.] Merr.), and sorghum with cotton (*Gossypium hirsutum* L.) had a significant impact on suppressing the density and total dry biomass of purple nutsedge (*Cyperus rotundus* L.) [2].

Accordingly, there is no information available on the combined effects of intercropping systems, irrigation intervals, and weed competition on the growth, physiological parameters, cottonseed quality, and water use efficiency of two cotton varieties. Therefore, the objective of this study is to conduct a comprehensive analysis of these effects. By evaluating the response of these cotton cultivars to different levels of moisture and weed stresses under various production conditions, this research aims to provide insights into the most effective adaptive cultural management strategies for optimizing cotton productivity and quality.

## 2 Materials and methods

### 2.1 Experimental site

**Fig. 1:**
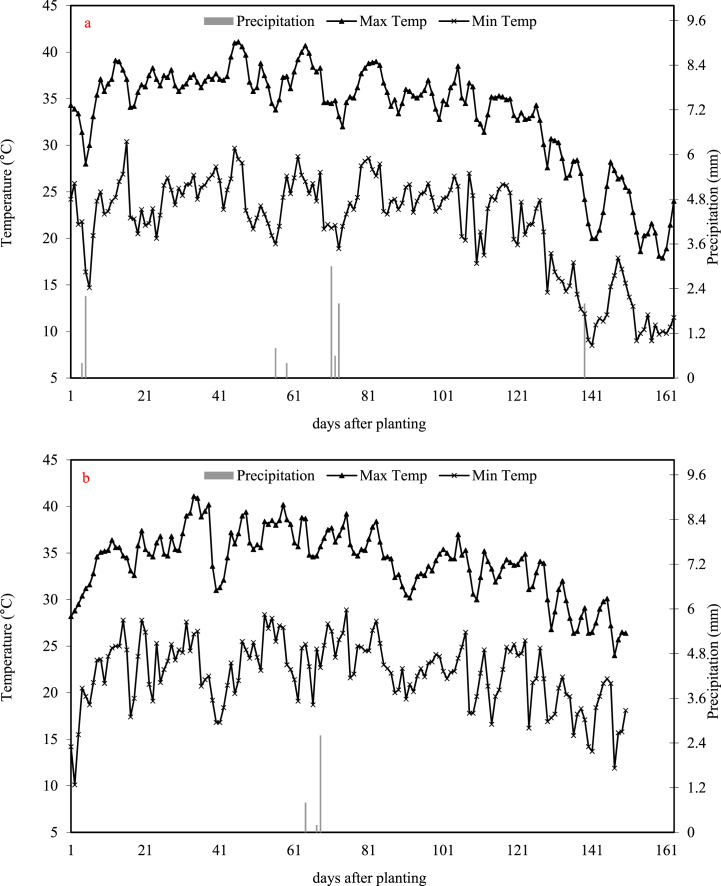
The minimum and maximum daily temperatures as well as precipitation during the period of cotton cultivation in Tehran for both a) 2021 and b) 2022.

A two-year field experiment was performed in 2021 and 2022 at the Faculty of Agriculture, Tarbiat Modares University, Tehran, Iran. The location of the study was at coordinates 35°44′N, 51°09′E, and an elevation of 1265 m above sea level. This area has a climate that ranges from arid to semi-arid, as classified by the Köppen climate classification system. The long-term average annual rainfall in this region is 233 mm, and the average temperature is 17.6 °C. Daily weather data, was from a Chitgar weather station, about 1 km away from the experimental site, with coordinates 35°44′N, 51°10′E, and an elevation of 1305 m above sea level. Fig. 1 reports the meteorological data recorded during the experiment in each year. The soil in the experimental area had a sandy loam texture. Table 1 presents the physical and chemical properties of three soil layers within the experiment. Additionally, the study site contained a diverse population of naturally occurring weeds, consisting of seven species (Table 2), with an overall density ranging from 157 to 857 seedlings m^−2^. The weed density was measured one month after planting. In the second year, the experiment was repeated on the same land.

**Table 1:** Physico-chemical properties of different layers of the experimental soil before the beginning of experiment.

Soil parameters	Soil depth (cm)		
	0–20	20–40	40–60
Sand (%)	64	68	66
Silt (%)	20	18	18
Clay (%)	16	14	16
Bulk density (g cm^−3^)	1.20	1.40	1.48
FC (0.033 MPa, % by wt.)	15.38	19.53	14.89
PWP (1.5 MPa, % by wt.)	7.19	8.39	6.26
Organic C (%)	1.79	1.56	1.09
pH	7.74	7.74	7.74
EC (dS m^−1^)	1.3	1.3	1.3
Available N (kg ha^−1^)	29.0	34.1	43.3
Available P (kg ha^−1^)	196	227	214
Available K (kg ha^−1^)	2085	2304	2466

The soil characteristics were determined according to Tandon [26].

FC (field capacity) and PWP (permanent wilting point) were determined according to Al-Rumikhani [40].

**Table 2:** The average weed proportions based on total dry weight measured in plots containing cotton, *Nepeta crispa*, and dragon's head in 2021 and 2022.

Latin name	Family	Bayer Code	Photosynthetic pathway	Proportion (%) in 2021	Proportion (%) in 2022
*Datura stramonium* L.	Solanaceae	DATST	C3	13.17	15.40
*Amaranthus retroflexus* L.	Amaranthaceae	AMARE	C4	23.15	20.23
*Xanthium strumarium* L.	Asteraceae	XANST	C3	34.41	21.08
*Tribulus terrestris* L.	Zygophyllaceae	TRBTE	C3	6.66	5.19
*Portulaca oleracea* L.	Portulacaceae	POROL	C4 or CAM	15.09	21.98
*Setaria viridis* (L.) P. Beauv.	Poaceae	SETVI	C4	3.87	13.36
*Sorghum halepense* (L.) Pers.	Poaceae	SORHA	C4	4.05	3.10
		Total dry weight (g m^−2^)		251.76	130.83

### 2.2 Experimental design and details

The experiment used a factorial arrangement within a randomized complete block design with three replications. The experiment consisted of four intercropping systems: mono-cropped cotton varieties 1) Golestan and 2) Hekmat, intercropped with 3) *Nepeta crispa* in 2021, and 4) dragon's head in 2022. There were three irrigation intervals of 3, 6, and 9 days, and two competition conditions of weed-free and weedy plots.

The intercropping system was implemented using a row-replacement series design in which alternating rows of cotton and *Nepeta crispa* (in 2021) or dragon's head (in 2022) were planted in a 1:1 ratio. The distance between plots was 1.5 m in this study. Each plot consisted of eight rows, each 6 m long. The rows were spaced 50 cm apart, and the distance between plants within the rows was 20 cm. The total area of each plot was 24 m^2^, with dimensions of 6 m in length and 4 m in width.

Before implementing the irrigation system in the field, all the necessary calculations were conducted based on the soil and plant characteristics. The drip irrigation system is comprised of two parts: a control unit and distribution lines. Inside the control unit, there was a hydrocyclone, disk filter, and control valves. The distribution system employed polythene (PE) pipes with diameters of 63 mm and 40 mm for the main and submain lines, respectively. The PE lateral lines were equipped with inline compensating drippers that had a discharge rate of 1.6 L per hour at a pressure of 1 bar. To accommodate the spacing between cotton plants within the rows, the dripper spacing was selected to be 20 cm. In the experiment, each row of crop was assigned one drip line. During each irrigation, the volume of water used for irrigation was determined by comparing the soil's moisture content to 100% field capacity. To measure the moisture content of the soil in each area, a TRIME-FM TDR (Time Domain Reflectometry, IMKO Micromodultechnik, Ettlingen, Germany) was utilized. The TDR device was calibrated to accurately measure soil water content over various ranges. In both years, access tubes were placed at the center of each plot to measure the soil water content. The TDR device was calibrated on-site, and the readings obtained were converted into volumetric soil water content. To maintain the consistency and simplicity of our irrigation treatments and avoid confounding factors, we adjusted the irrigation amount for each plot based on the soil moisture content measured by the TDR tubes.

To prepare the land, moldboard plowing tilled the soil to a depth of 30 cm, followed by disking. The seeds were planted by hand at a depth of 1–2 cm on June 15th and May 26th, 2021 and 2022, respectively. The seed planting rate was 30 kg/ha (c.a., 300,000 seeds ha^−1^), and excess plants were removed at the 4-leaf stage to ensure the desired density (100,000 plants ha^−1^).

Irrigation began immediately after planting, with the first irrigation applied right after sowing the seeds. Surface drip irrigation was used, and the plots were irrigated according to the designated irrigation treatments until maturity. In 2021, the volume of irrigation water applied was 9873, 6100, and 4650 m^3^ ha^−1^ for irrigation intervals of 3, 6, and 9 days, respectively. In 2022, the volumes were 9071, 5605, and 4272 m^3^ ha^−1^ for the corresponding irrigation intervals.

No pesticides were used in this study. A total of 150 kg nitrogen ha^−1^ was applied manually to the soil surface as urea (46% N) in three equal splits at specific growth stages of the cotton plants. The stages were the leaf development stage (growth stage BBCH 10–19), the start of inflorescence emergence (growth stage BBCH 51), and the end of flowering (growth stage BBCH 69). The surface drip irrigation immediately dissolved the fertilizer after application.

In weed-free plots, manual weeding was performed whenever necessary. The plants were harvested on September 20th and 21st, 2021 and 2022, respectively.

### 2.3 Dependent variables

The agronomic characteristics, such as 1000-seed weight, number of bolls per plant, plant height, cottonseed protein content, and cottonseed oil content, were randomly assessed from five mature plants in each plot. The measurements for leaf greenness index, photosynthetic rate, and leaf area were taken during full flowering. The total dry weight, cottonseed yield, lint yield, and seed cotton yield were calculated based on a harvested area of 2 m^2^ in the central rows. The seed cotton yield was determined at a moisture content of 12%. For measuring the photosynthetic rate, the upper three leaves of the plants were evaluated using a portable gas exchange system (Li-Cor 6400, Li-Cor Inc., Lincoln, NE, USA). The measurement conditions inside the chamber included a leaf temperature of 35 °C, reference CO_2_ concentration of 480 μmol mol^−1^, reference H_2_O concentration of 1 mmol mol^−1^, and a photosynthetically active radiation (PAR) of 1800 μmol m^−2^ s^−1^. The IRGA (infra-red gas analyzer) was calibrated manually, and the levels of reference CO_2_ and reference H_2_O were stabilized before taking measurements. Leaf greenness was estimated using a SPAD-502 device (Konica Minolta, Osaka, Japan), and leaf area was measured using a leaf area meter (Delta-T area meter; Delta-T Devices Ltd., Cambridge, UK). The leaf area index (LAI) was calculated by dividing the leaf area by the ground area. The total dry weight of each weed species was determined within a harvested area of 1 m^2^ during full flowering of cotton. All plant organ samples were dried at 70 °C for 48 h then weighed. The harvest index (HI) was computed by dividing the economic yield by the aboveground biomass for cottonseed yield, lint yield, and seed cotton yield. Water use efficiency (WUE) was calculated by dividing the seed cotton yield by the volume of water applied. To determine the total oil concentration of the cottonseed, a Soxhlet extraction method was employed [25]. This involved packing 5 g of milled seeds in a paper extraction and extracting the oils using 300 ml of petroleum benzene in a Soxhlet extractor for 4 h. The oil concentration, based on the whole seed, was expressed as a percentage (g 100 g^−1^). After wet digestion, total Kjeldahl nitrogen was measured by the method described by Tandon [26], and the nitrogen percentage was multiplied by 6.25 to obtain the protein percentage (g 100 g^−1^).

### 2.4 LER (land equilibrium ratio) assessment

According to Beets [27], the LER values were evaluated using the following equation:

LER = Y_icotton_/ Y_mcotton_ + Y_imp_/Y_mmp_

where Y_mcotton_ and Y_icotton_ represent the seed cotton yield in mono-cropped and inter-cropped cotton varieties, respectively, and Y_mmp_ and Y_imp_ are the total dry weight of *Nepeta crispa* in 2021 or the seed yield of dragon's head in 2022 in mono-cropped and inter-cropped systems, respectively. When the LER value equals 1.0, it indicates equal competitive ability between the two crops in intercropping. This means that the inter-specific competition is equivalent to the intra-species competition within the intercropping components. Additionally, if an increase in the yield of one crop coincides with a decrease in the yield of another crop in intercropping, the LER value will be equal to 1.0. If the LER value is less than 1.0, it suggests that mono-cropping is more advantageous compared to intercropping. Conversely, if the LER value is greater than 1.0, intercropping is considered more beneficial than mono-cropping.

### 2.5 Statistical analysis

The main effects of three experimental factors and their interactions were examined through analysis of variance (ANOVA) using the general linear model (GLM) procedure in Statistical Analysis System (SAS) software version 9.0. To assess the significance, the total dry weight of weeds was included as a covariate, but it was determined to be non-significant (*P* > 0.05). The main effects of intercropping systems, irrigation intervals, competition conditions, and their two- and three-way interactions were treated as fixed effects. The normality of residuals was tested using the UNIVARIATE procedure. To compare means, the protected least significant difference (protected LSD) test was applied at a significance level of *P* ≤ 0.05. Principal components analysis was conducted using the Microsoft Excel XLSTAT program (Version 2019.2.2.59614).

## 3 Results

### 3.1 Weeds frequency

In this experiment, over two years, we observed the prevalence of broad-leaved weeds in the field. The dominant weed species encountered were *Xanthium strumarium*, *Amaranthus retroflexus*, and *Portulaca oleracea*. Additionally, we noted the presence of *Datura stramonium*, *Tribulus terrestris*, *Sorghum halepense*, and *Setaria viridis* (Table 1). Interestingly, in the second year, there was a noticeable increase in the proportion of weed populations that exhibited the C4 photosynthetic pathway compared to the first year. This increase amounted to approximately 13%. Furthermore, there were some intriguing variations when comparing the specific weed species between the two years. In 2021, *Xanthium strumarium* accounted for about 34% of the total weed population, which was higher than its proportion in 2022. Conversely, the proportions of *Portulaca oleracea* and *Setaria viridis* were higher in 2022 when compared to their respective proportions in 2021. Specifically, *Portulaca oleracea* constituted around 22% of the weed population in 2022, while *Setaria viridis* exhibited a significant increase of 245% compared to the previous year (Table 1).

### 3.2 Analysis of variance

The analysis of variance revealed significant effects of intercropping systems, irrigation intervals, competition conditions, and their two- and three-way interactions on a majority of traits (Table 3, Table 4, Table 5, Table 6, Table 7, Table 8). It is important to note that the responses of the measured traits varied in this experiment based on the different experimental factors. Therefore, we conducted mean comparisons in a specific sequence: first, we considered the significance of the three-way interaction; then, we proceeded to analyze the two-way interactions; finally, we examined the main effects. This approach ensured that when the three-way interaction was found to be significant, we avoided making mean comparisons for the two-way interactions and main effects.

**Table 3:** Analysis of variance (mean square) of the main effects, two-way, and three-way interactions of intercropping systems (four growth conditions including mono- and inter-cropped cotton varieties Golestan and Hekmat), irrigation (three intervals of 3, 6, and 9 days), and competition (weed-free and weedy plots) on the total dry weight, economic yields, and harvest indices of cotton in 2021.

Source of variation	DF	Total dry weight	Cottonseed yield	Lint yield	Seed cotton yield	Seed cotton HI	Cottonseed HI	Lint HI
Block	2	447773^ns^	49792^ns^	7023^ns^	77736^ns^	12.90^ns^	6.46^ns^	4.09^ns^
Intercropping (IC)	3	37575788**	2573815**	1119874**	7047269**	23.23^ns^	29.29*	4.91^ns^
Irrigation (I)	2	13529922**	225966*	93276^ns^	607471^ns^	587.84**	183.45**	114.75**
IC × I	6	1224677^ns^	35782^ns^	20345^ns^	97967^ns^	20.44^ns^	16.60^ns^	0.57^ns^
Competition (C)	1	57485923**	3644726**	1512001**	9851758**	51.74^ns^	10.60^ns^	15.50^ns^
IC × C	3	2435848^ns^	186786^ns^	79737^ns^	510174^ns^	32.04^ns^	15.56^ns^	5.17^ns^
I × C	2	6195188**	369092**	230044*	1177034**	41.79^ns^	18.36^ns^	9.01^ns^
IC × I × C	6	1356031^ns^	142886^ns^	59024^ns^	368855^ns^	80.40**	50.92**	6.43^ns^
Error	46	905024	69747	47256	221585	18.03	8.59	5.87
CV (%)	–	25.00	25.90	32.01	27.71	9.33	10.70	13.37

HI: harvest index; DF: degree of freedom; CV: coefficient of variation; ns: *P* > 0.05; *: *P* ≤ 0.05; **: *P* ≤ 0.01.

**Table 4:** Analysis of variance (mean square) of the main effects, two-way, and three-way interactions of intercropping systems (four growth conditions including mono- and inter-cropped cotton varieties Golestan and Hekmat), irrigation (three intervals of 3, 6, and 9 days), and competition (weed-free and weedy plots) on the total dry weight, economic yields, and harvest indices of cotton in 2022.

Source of variation	DF	Total dry weight	Cottonseed yield	Lint yield	Seed cotton yield	Seed cotton HI	Cottonseed HI	Lint HI
Block	2	4722219*	148235*	78613^ns^	441296*	117.30*	33.26^ns^	31.22**
Intercropping (IC)	3	28065327**	925613**	677167**	3108120**	141.37**	79.21**	36.16**
Irrigation (I)	2	14178850**	426054**	505430**	1849502**	50.10^ns^	60.72*	27.56*
IC × I	6	1989467^ns^	49912^ns^	39640^ns^	161389^ns^	57.75^ns^	35.26*	22.13**
Competition (C)	1	60351591**	1226504**	1134470**	4720154**	1239.72**	599.04**	115.23**
IC × C	3	640707^ns^	21039^ns^	41269^ns^	118568^ns^	73.08^ns^	31.41^ns^	10.43^ns^
I × C	2	8467352**	315521**	264738**	1157803**	14.60^ns^	1.95^ns^	8.58^ns^
IC × I × C	6	1030619^ns^	60943^ns^	37963^ns^	160052^ns^	25.77^ns^	47.81**	8.20^ns^
Error	46	1126477	37114	25619	117486	26.20	12.88	5.99
CV (%)	–	32.94	26.51	29.84	27.14	12.17	14.57	14.06

HI: harvest index; DF: degree of freedom; CV: coefficient of variation; ns: *P* > 0.05; *: *P* ≤ 0.05; **: *P* ≤ 0.01.

**Table 5:** Analysis of variance (mean square) of the main effects, two-way, and three-way interactions of intercropping systems (four growth conditions including mono- and inter-cropped cotton varieties Golestan and Hekmat), irrigation (three intervals of 3, 6, and 9 days), and competition (weed-free and weedy plots) on the 1000-seed weight (TSW), number of bolls per plant, plant height, and leaf area index (LAI) of cotton in 2021.

Source of variation	DF	TSW	Bolls no plant^−1^	Plant height	LAI
Block	2	1460.25**	8.04^ns^	91.72^ns^	0.205^ns^
Intercropping (IC)	3	81.47^ns^	40.70^ns^	829.69**	3.850**
Irrigation (I)	2	117.25^ns^	360.13**	533.72*	18.504**
IC × I	6	314.86^ns^	18.27^ns^	216.91^ns^	0.892^ns^
Competition (C)	1	2350.69**	854.22**	34409.39**	154.445**
IC × C	3	43.43^ns^	56.26^ns^	176.65^ns^	0.182^ns^
I × C	2	106.99^ns^	76.51^ns^	894.06**	5.004**
IC × I × C	6	139.77^ns^	56.88^ns^	192.65^ns^	0.650^ns^
Error	46	219.46	26.23	167.95	0.399
CV (%)	–	20.09	38.90	15.01	24.32

DF: degree of freedom; CV: coefficient of variation; ns: *P* > 0.05; *: *P* ≤ 0.05; **: *P* ≤ 0.01.

**Table 6:** Analysis of variance (mean square) of the main effects, two-way, and three-way interactions of intercropping systems (four growth conditions including mono- and inter-cropped cotton varieties Golestan and Hekmat), irrigation (three intervals of 3, 6, and 9 days), and competition (weed-free and weedy plots) on the 1000-seed weight (TSW), number of bolls per plant, plant height, and leaf area index (LAI) of cotton in 2022.

Source of variation	DF	TSW	Bolls no plant^−1^	Plant height	LAI
Block	2	176.17*	4.63^ns^	1514.43**	0.253^ns^
Intercropping (IC)	3	12.60^ns^	28.46*	276.65^ns^	16.668**
Irrigation (I)	2	145.34^ns^	240.29**	782.72*	26.208**
IC × I	6	182.10**	11.31^ns^	351.98^ns^	4.115**
Competition (C)	1	1975.06**	533.56**	1922.00**	82.303**
IC × C	3	13.75^ns^	5.96^ns^	138.89^ns^	11.916**
I × C	2	130.73^ns^	127.10**	121.17^ns^	15.823**
IC × I × C	6	221.75**	7.00^ns^	207.28^ns^	2.995**
Error	46	54.08	9.02	200.53	0.126
CV (%)	–	11.53	32.46	26.08	21.31

DF: degree of freedom; CV: coefficient of variation; ns: *P* > 0.05; *: *P* ≤ 0.05; **: *P* ≤ 0.01.

**Table 7:** Analysis of variance (mean square) of the main effects, two-way, and three-way interactions of intercropping systems (four growth conditions including mono- and inter-cropped cotton varieties Golestan and Hekmat), irrigation (three intervals of 3, 6, and 9 days), and competition (weed-free and weedy plots) on the leaf greenness index, photosynthetic rate, contents of cottonseed protein and oil, and water use efficiency (WUE) of cotton in 2021.

Source of variation	DF	Greenness index	Photosynthetic rate	Protein content	Oil content	WUE
Block	2	6.19^ns^	4.15^ns^	3.75^ns^	0.018^ns^	0.001^ns^
Intercropping (IC)	3	77.03^ns^	4.76^ns^	2.90^ns^	0.037^ns^	0.144**
Irrigation (I)	2	172.92^ns^	298.73**	5.22^ns^	1.342**	0.151**
IC × I	6	32.24^ns^	2.69^ns^	1.99^ns^	0.043^ns^	0.006^ns^
Competition (C)	1	7017.18**	135.93**	479.94**	0.129*	0.192**
IC × C	3	106.53^ns^	16.27^ns^	3.98^ns^	0.023^ns^	0.007^ns^
I × C	2	329.16**	6.04^ns^	6.18^ns^	0.259**	0.003^ns^
IC × I × C	6	87.44^ns^	11.79^ns^	5.88^ns^	0.023^ns^	0.006^ns^
Error	46	60.51	10.35	3.41	0.028	0.004
CV (%)	–	16.52	29.75	7.00	0.77	23.73

DF: degree of freedom; CV: coefficient of variation; ns: *P* > 0.05; *: *P* ≤ 0.05; **: *P* ≤ 0.01.

**Table 8:** Analysis of variance (mean square) of the main effects, two-way, and three-way interactions of intercropping systems (four growth conditions including mono- and inter-cropped cotton varieties Golestan and Hekmat), irrigation (three intervals of 3, 6, and 9 days), and competition (weed-free and weedy plots) on the leaf greenness index, photosynthetic rate, contents of cottonseed protein and oil, and water use efficiency (WUE) of cotton in 2022.

Source of variation	DF	Greenness index	Photosynthetic rate	Protein content	Oil content	WUE
Block	2	697.81**	40.66^ns^	8.97**	2.15^ns^	0.017**
Intercropping (IC)	3	931.06**	7.40^ns^	8.06**	10.31^ns^	0.079**
Irrigation (I)	2	352.58^ns^	104.80**	1.12^ns^	16.97^ns^	0.024**
IC × I	6	230.20^ns^	9.50^ns^	2.16^ns^	19.35^ns^	0.001^ns^
Competition (C)	1	3771.46**	608.55**	32.53**	139.90**	0.085**
IC × C	3	130.75^ns^	6.48^ns^	0.44^ns^	3.75^ns^	0.002^ns^
I × C	2	16.94^ns^	20.73^ns^	2.27^ns^	10.30^ns^	0.009^ns^
IC × I × C	6	237.48^ns^	17.23^ns^	0.86^ns^	18.99^ns^	0.003^ns^
Error	46	136.72	21.14	1.50	13.47	0.003
CV (%)	–	31.20	34.50	4.35	18.50	26.02

DF: degree of freedom; CV: coefficient of variation; ns: *P* > 0.05; *: *P* ≤ 0.05; **: *P* ≤ 0.01.

### 3.3 Total dry weight, economic yields, and harvest indices

**Fig. 2:**
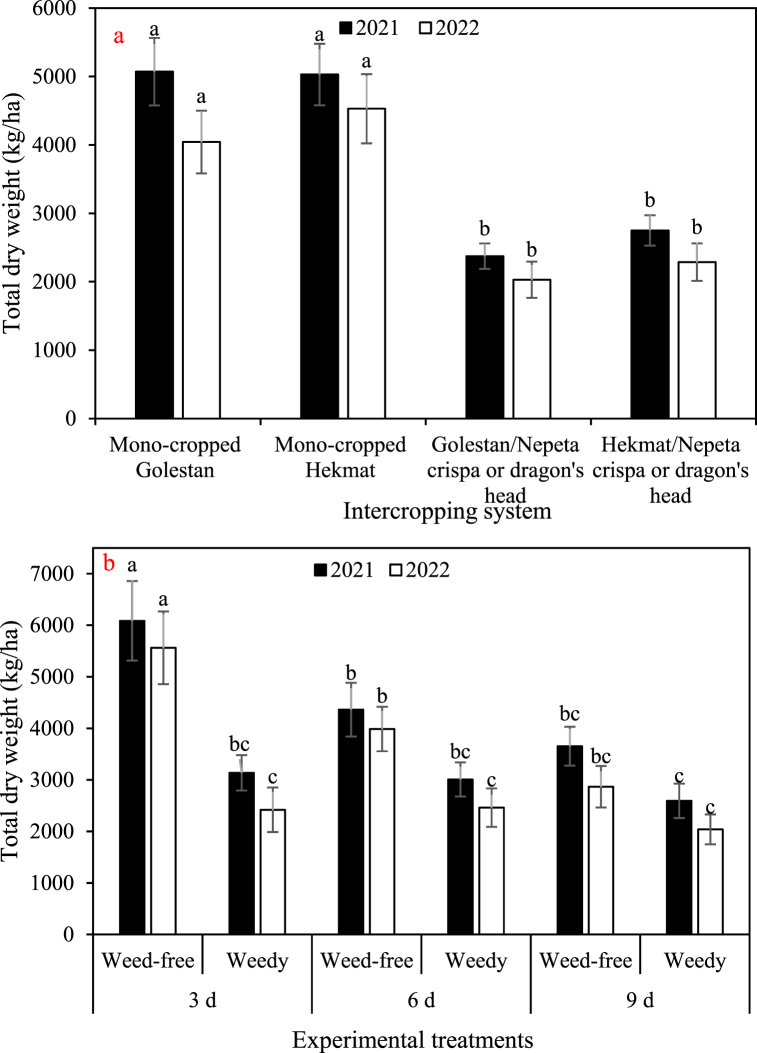
a) The main effects of intercropping systems (four growth conditions including mono- and inter-cropped cotton varieties Golestan and Hekmat), and b) two-way interactions of irrigation (three intervals of 3, 6, and 9 days), and competition (weed-free and weedy plots) on the total dry weight of cotton in 2021 and 2022. In each year, different letters denoted above the columns indicate statistically significant differences at a significance level of *P* ≤ 0.05.

**Fig. 3:**
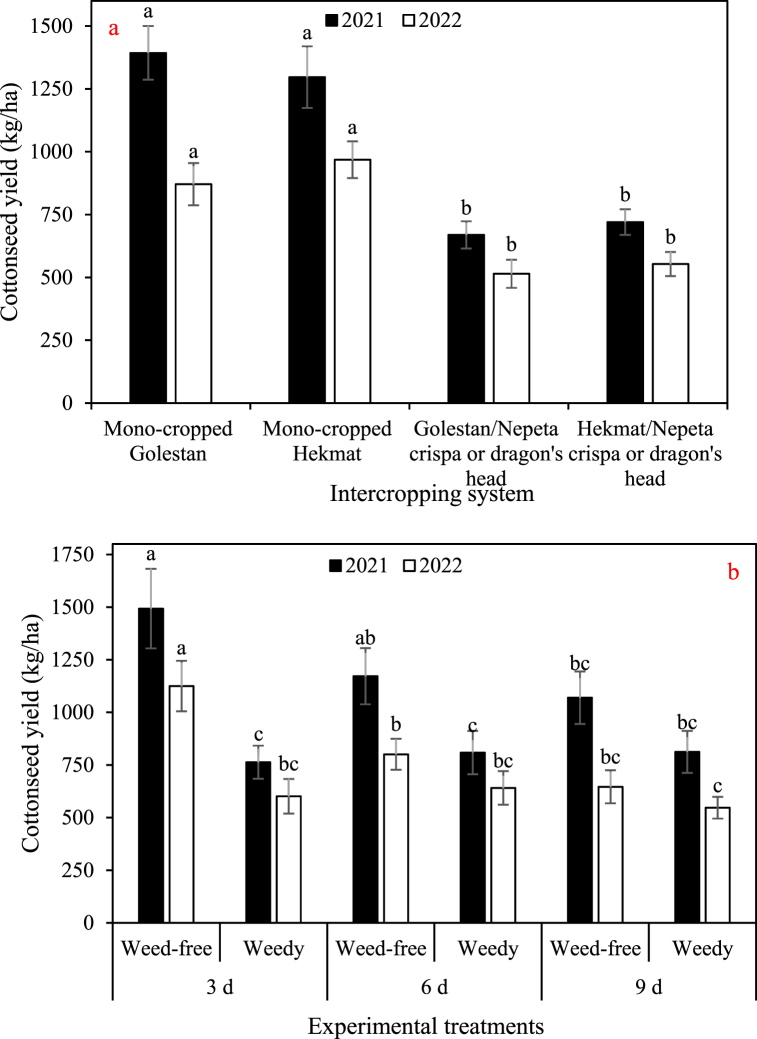
a) The main effects of intercropping systems (four growth conditions including mono- and inter-cropped cotton varieties Golestan and Hekmat), and b) two-way interactions of irrigation (three intervals of 3, 6, and 9 days), and competition (weed-free and weedy plots) on the cottonseed yield of cotton in 2021 and 2022. In each year, different letters denoted above the columns indicate statistically significant differences at a significance level of *P* ≤ 0.05.

**Fig. 4:**
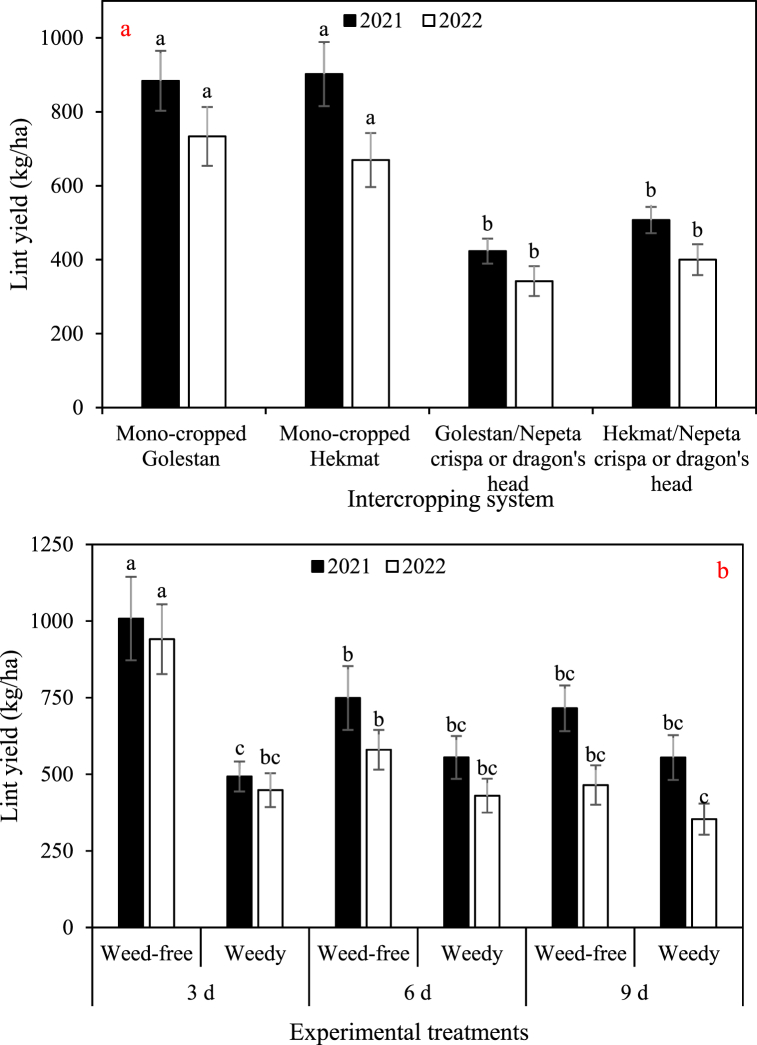
a) The main effects of intercropping systems (four growth conditions including mono- and inter-cropped cotton varieties Golestan and Hekmat), and b) two-way interactions of irrigation (three intervals of 3, 6, and 9 days), and competition (weed-free and weedy plots) on the lint yield of cotton in 2021 and 2022. In each year, different letters denoted above the columns indicate statistically significant differences at a significance level of *P* ≤ 0.05.

**Fig. 5:**
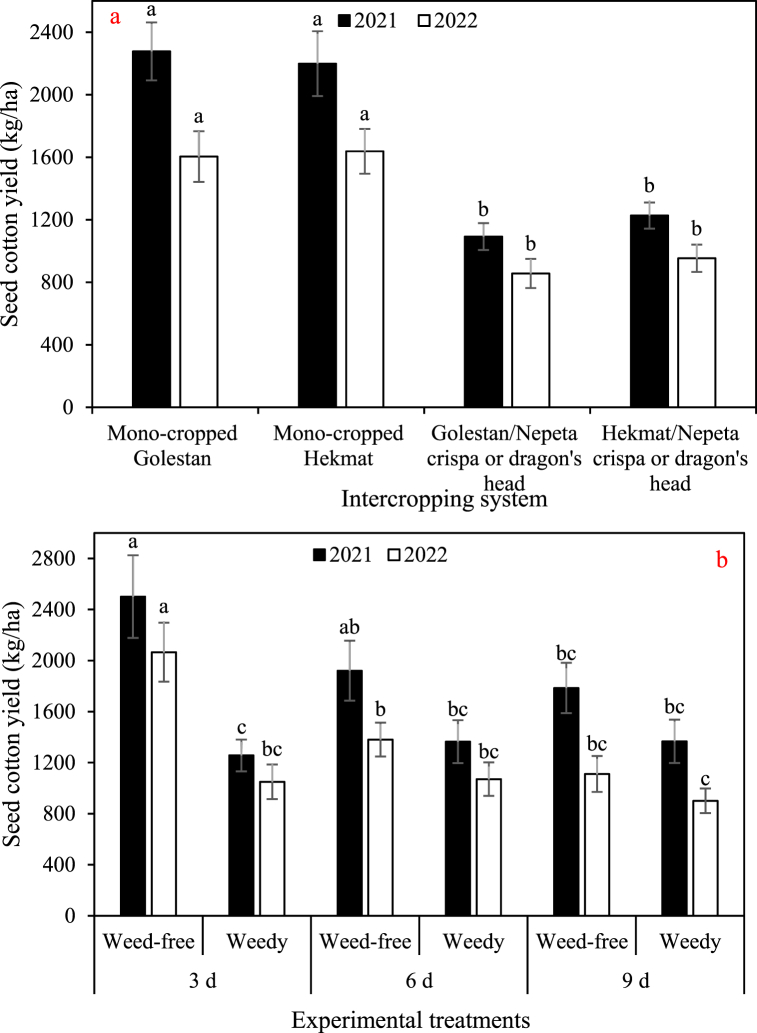
a) The main effects of intercropping systems (four growth conditions including mono- and inter-cropped cotton varieties Golestan and Hekmat), and b) two-way interactions of irrigation (three intervals of 3, 6, and 9 days), and competition (weed-free and weedy plots) on the seed cotton yield of cotton in 2021 and 2022. In each year, different letters denoted above the columns indicate statistically significant differences at a significance level of *P* ≤ 0.05.

The data depicted in Fig. 2, Fig. 3, Fig. 4, Fig. 5 demonstrate a consistent trend among the total dry weight, cottonseed yield, lint yield, and seed cotton yield in response to the experimental factors. The results showcased a remarkable similarity in their overall patterns. On average, when comparing the different treatments, the data clearly indicates that the weights of total biomass, cottonseed, lint, and seed cotton were significantly higher in 2021 compared to 2022. Specifically, there was an observed increase of 18% in total biomass, a substantial 40% increase in cottonseed weight, a notable 27% increase in lint weight, and a significant 35% increase in seed cotton weight in 2021 as compared to 2022. There was no significant difference observed between the Golestan and Hekmat varieties, whether they were grown in mono-crop or intercropped with *Nepeta crispa* and dragon's head (*Lallemantia iberica*), with regards to these specific traits. The values for these traits were observed to decrease with an increase in irrigation interval and an increase in weed presence. Among the four traits, weeds had the most significant impact on total dry weight. Weed control increased total dry weight by 1.4-fold (in irrigation intervals of 9 days in both years) to 2.3-fold (in irrigation intervals of 3 days in 2022). On the other hand, weeds had the least impact on cottonseed yield, resulting in damage ranging from 18% (in irrigation intervals of 9 days in 2022) to 96% (in irrigation intervals of 3 days in 2021). In plots where weeds were effectively controlled, the results showed consistent trends across two years. Increasing the irrigation intervals from 3 days to 6 days and 9 days resulted in substantial reductions in various parameters. Specifically, there was a significant decrease in total dry weight, with reductions of 39% and 80% observed for the respective intervals. Similarly, cottonseed yield exhibited decreases of 34% and 57%, while lint yield decreased by 48% and 72%. The overall seed cotton yield also experienced reductions, with decreases of 40% and 63% for the 6-day and 9-day intervals, respectively. Conversely, in plots where weeds were allowed to grow, the impact of increased irrigation intervals differed somewhat. Across two years, changing the irrigation intervals from 3 days to 6 days and 9 days had more nuanced effects. While there was only a minor 1% decrease in total dry weight for the 6-day interval, the 9-day interval saw a more substantial 20% decrease. Interestingly, the effect on cottonseed yield varied depending on the irrigation interval. The 6-day interval led to a modest increase of 6% in cottonseed yield, whereas the 9-day interval resulted in a slight decline of 10%. Similarly, the lint yield response was divergent, with the 6-day interval showing a small increase of 4%, while the 9-day interval exhibited an 8% decrease. Finally, the seed cotton yield displayed mixed outcomes, with a 5% increase for the 6-day interval and a 4% decrease for the 9-day interval. These findings underscore the importance of weed control and appropriate irrigation management in optimizing crop productivity. Effective weed management can mitigate yield losses, while careful consideration of irrigation intervals is crucial to maintaining optimal growing conditions and maximizing overall crop performance.

The harvest indices of cotton, which were calculated based on seed cotton yield, cottonseed yield, and lint yield, exhibited distinct responses to the experimental factors as shown in (Supplementary Figs. S1–S3). Interestingly, weedy plots, longer irrigation intervals, and intercropped growth conditions consistently yielded higher values for harvest indices. Specifically, the harvest indices for seed cotton, cottonseed, and lint ranged from 35.3% to 56.5%, 18.3%–35.0%, and 15.4%–20.5%, respectively (Supplementary Figs. S1–S3).

### 3.4 Yield components and morphological traits

In the first year, the impact of competition on 1000-seed weight was found to be significant (Supplementary Fig. S4). Weeds were responsible for a 17% decrease in the 1000-seed weight. Moving on to the second year, a three-way interaction between intercropping systems, irrigation, and competition significantly influenced this trait (Supplementary Fig. S4). Furthermore, weeds consistently led to a reduction in 1000-seed weight across all irrigation and intercropping conditions in the second year. Notably, the highest 1000-seed weight, approximately 80 g, was observed in the intercropping systems, while the effect of irrigation on this trait did not exhibit a clear trend (Supplementary Fig. S4).

The effects of irrigation and weed on the number of bolls per plant were found to be significant in the first year (Supplementary Fig. S5). As the irrigation intervals increased from 3 days to 6 days and 9 days, the number of bolls per plant decreased by 19%–85%. Additionally, weeds were found to have a detrimental effect, reducing the number of bolls per plant by 71%. In the second year, the intercropping system and the interaction between irrigation and compaction had significant effects on this trait (Supplementary Fig. S5). The cotton variety Hekmat intercropped with dragon's head exhibited the highest value for the number of bolls per plant. Specifically, an irrigation interval of 3 days in weed-free conditions resulted in the highest number of bolls per plant, with a total of 18 bolls observed (Supplementary Fig. S5).

The tallest plants were consistently observed in intercropping systems, reaching a height of approximately 92 cm in the first year. In comparison, weed-free plots that received irrigation every 3 days exhibited a greater height of around 120 cm in the same year. However, in the second year, the plants in plots without weeds and irrigated every 3 days exhibited a reduced height of approximately 60 cm. The results indicate that overall plant height decreased with an increase in both the irrigation interval and the presence of weeds (Supplementary Fig. S6). These findings suggest that drought and weed competition can have a detrimental effect on the growth and development of the plants, ultimately stunting their height.

**Fig. 6:**
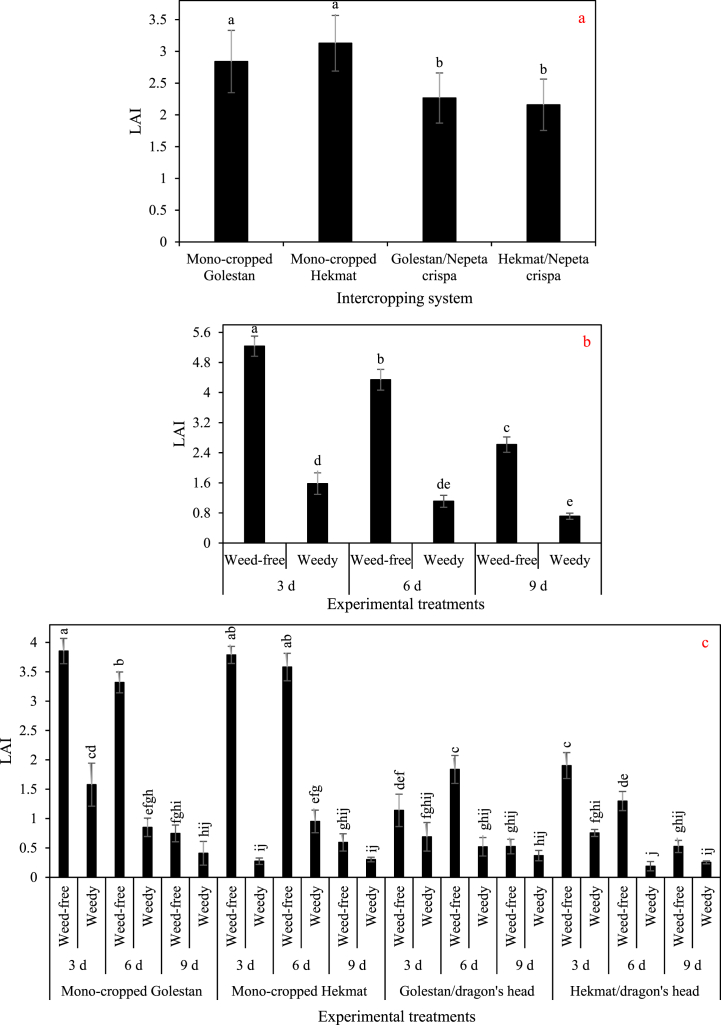
a) The main effect of intercropping systems and b) the two-way interaction of irrigation and competition in 2021, and c) the three-way interaction of intercropping systems (four growth conditions including mono- and inter-cropped cotton varieties Golestan and Hekmat), irrigation (three intervals of 3, 6, and 9 days), and competition (weed-free and weedy plots) on the leaf area index (LAI) of cotton in 2022. In each year, different letters denoted above the columns indicate statistically significant differences at a significance level of *P* ≤ 0.05.

The leaf area index (LAI) of cotton varieties did not exhibit significant differences under all conditions (Fig. 6). However, it is noteworthy that in both years, the LAI was observed to decrease significantly due to two factors: reduced irrigation frequency and increased weed competition.

### 3.5 Physiological and seed quality traits

The leaf greenness index of cotton showed a significant increase with higher irrigation frequencies under weed-free conditions (Supplementary Fig. S7). It is worth mentioning that the intercropping system has an impact on this particular trait. In the case of mono-cropped Golestan, as well as intercropped Hekmat, the highest leaf greenness index was observed (Supplementary Fig. S7).

**Fig. 7:**
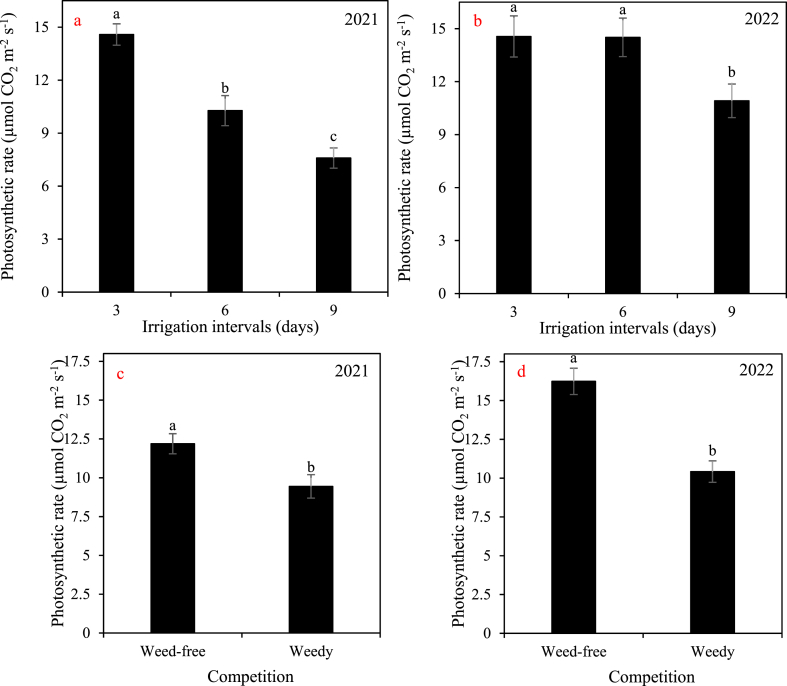
The main effects of a and b) irrigation (three intervals of 3, 6, and 9 days), c and d) competition (weed-free and weedy plots) on the photosynthetic rate of cotton in 2021 and 2022. In each year, different letters denoted above the columns indicate statistically significant differences at a significance level of *P* ≤ 0.05.

In the first year, extending the irrigation interval from 3 days to 6 days and 9 days resulted in a substantial decrease in the photosynthetic rate, ranging from 42% to 92% (Fig. 7). However, in the second year, the reduction in photosynthetic rate due to increased irrigation intervals was relatively lower, ranging from 0.03% to 33% (Fig. 7). Comparing weedy plots to weed-free plots, the plant photosynthesis rate showed a significant difference. Specifically, in 2021, the photosynthesis rate in weedy plots was 29% lower than in weed-free plots, and in 2022, it was 56% lower (Fig. 7).

**Fig. 8:**
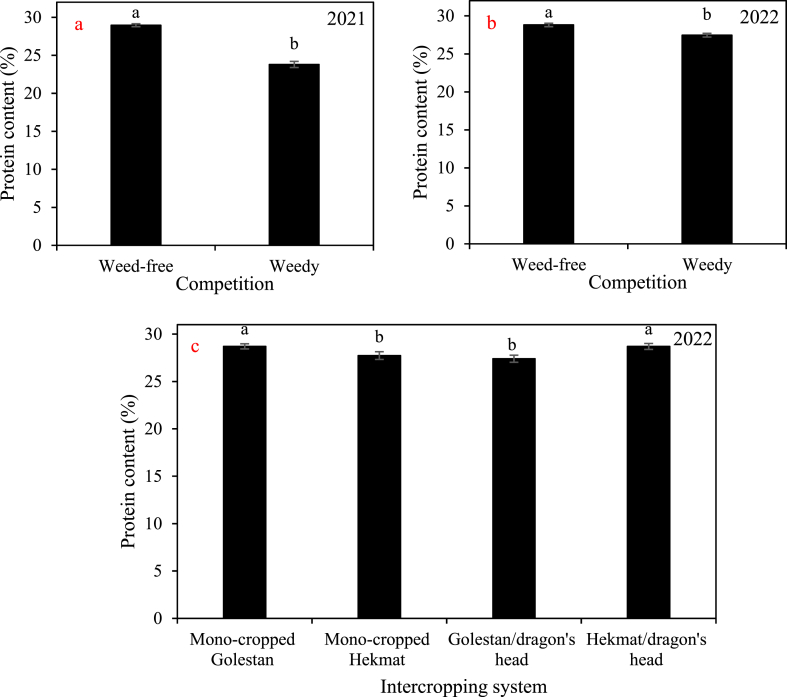
The main effects of a and b) competition (weed-free and weedy plots) and c) intercropping systems (four growth conditions including mono- and inter-cropped cotton varieties Golestan and Hekmat) on the cottonseed protein content of cotton in 2021 and 2022. In each year, different letters denoted above the columns indicate statistically significant differences at a significance level of *P* ≤ 0.05.

In both years, there was a significant decrease in seed protein content caused by weeds, resulting in a reduction ranging from 1.34% to 5.17% (Fig. 8). However, an interesting observation was made when intercropping the cotton variety Hekmat with dragon's head. It was found that this intercropping combination significantly led to an increase in seed protein content (Fig. 8).

**Fig. 9:**
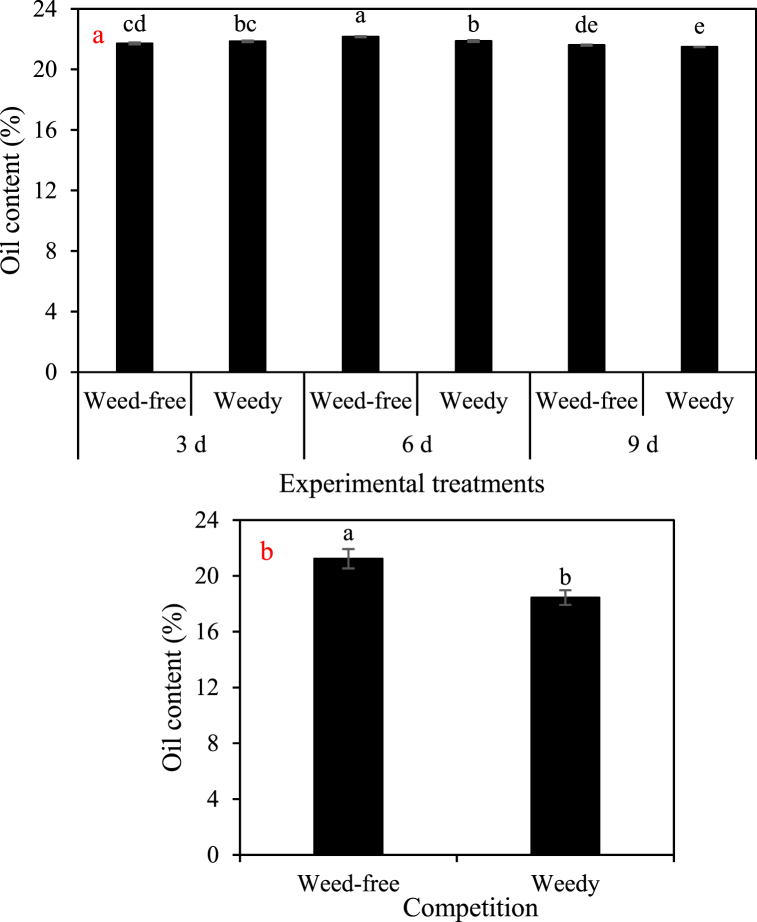
a) The two-way interaction of irrigation (three intervals of 3, 6, and 9 days) and competition (weed-free and weedy plots) in 2021 and b) the main effect of competition on the cottonseed oil content of cotton in 2022. In each year, different letters denoted above the columns indicate statistically significant differences at a significance level of *P* ≤ 0.05.

Moderate irrigation and weed-free conditions could lead to a significant increase in seed oil content, ranging from 0.67% to 2.78% (Fig. 9).

### 3.6 Principal components analysis

This study utilized principal component biplots to analyze the relationships among the different treatment combinations and the various parameters measured in cotton cultivation (Supplementary Fig. S8). In both years, the principal component analysis (PCA) revealed that the first two principal components (PCs) accounted for more than 70% of the total variation in the data. This indicates that these two components capture the majority of the variability present in the dataset. Furthermore, the variables "leaf area index" and "total dry weight" exhibited the highest positive vector loadings in PC1 for both years. This suggests that these variables have the most significant influence on PC1. By examining the biplots (Supplementary Fig. S8) and conducting squared cosine analysis between the variable vectors and the PC axes, treatment values greater than 0.5 were found. These values can be used to compare and rank the treatments based on their performance. In the first year, the order of treatments with values greater than 0.5 was 532 > 111 > 211 > 121 > 522 > 221 > 512 > 422 > 412 > 432. In the second year, the order of treatments with values greater than 0.5 was 111 > 532 > 211 > 412 > 432 > 221 > 121 > 132 > 422 > 522 > 232. Regarding the numbers, refer to Supplementary Fig. S8 for a visual representation of the treatments. Each digit of the number represents a specific treatment of the experiment. For instance, in the number 532, the digit 5 indicates the inter-cropped cotton variety Hekmat, the digit 3 represents irrigation at 9-day intervals, and the digit 2 signifies weedy plots. Based on the findings, it was observed that mono-cropped Golestan performed well under unstressed conditions such as interval irrigation of 3 days and weed-free conditions. On the other hand, intercropped Hekmat demonstrated better resilience to both moisture and weed stresses.

### 3.7 Water use efficiency and intercropping estimation

The experiment results (Supplementary Fig. S9) revealed that there was no significant difference in water use efficiency (WUE) among the different cotton varieties tested under all experimental conditions. However, it was observed that increasing the irrigation interval in both years led to an improvement in WUE, while the presence of weeds had a negative impact on WUE (Supplementary Fig. S9).

**Fig. 10:**
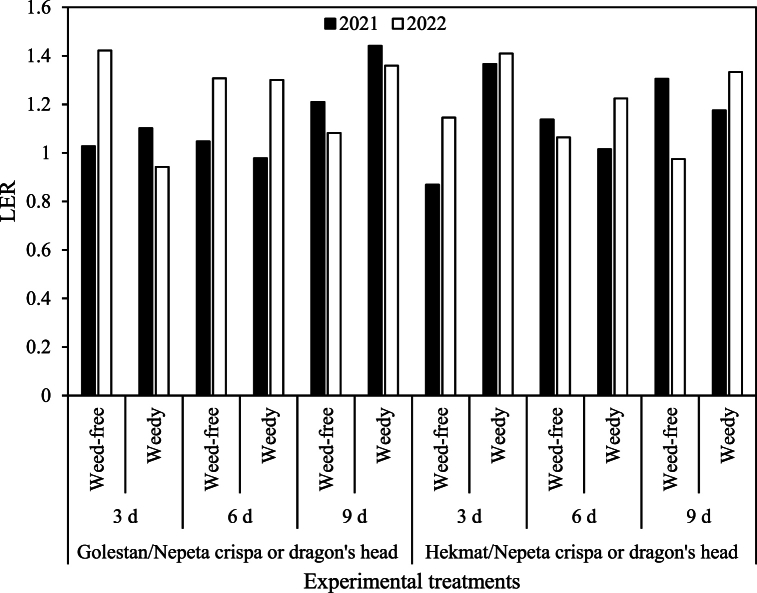
Effect of intercropped cotton varieties (Golestan and Hekmat) with *Nepeta crispa* (2021) or dragon's head (2022) on land equilibrium ratio (LER).

In both years, the LER (Land equivalent ratio) indices of both intercropping systems were generally favorable, indicating higher productivity compared to sole cropping. The only exception was observed in the intercropping system with cotton variety Hekmat/*Nepeta crispa*, which showed a LER index lower than 1.0 when irrigated every 3 days under weed-free conditions in 2021. Similarly, the intercropping system with Hekmat/dragon's head had a LER index below 1.0 when irrigated every 9 days under weed-free conditions in 2022 (Fig. 10). The intercropping systems consistently showed the highest LER indices under weedy conditions, highlighting the significance of intercropping as a valuable method in integrated weed management (IWM).

## 4 Discussion

Cotton, a vital industrial crop, poses significant agronomic challenges to producers, and there is a scarcity of data available, particularly in the context of sustainable agriculture. To date, there has been a lack of investigation regarding the combined effects of intercropping, irrigation, and weed competition on yield performance, morphological parameters, leaf gas exchange, greenness, cottonseed quality, and water use efficiency. However, given the significance of these factors, it is an area of great interest for research and exploration.

These results shed light on the dynamic nature of weed populations in the experimental field, highlighting shifts in species composition and changes in the prevalence of certain weed types over time. After analyzing the temperature and rainfall fluctuations, it became evident that the second year experienced significantly higher temperatures and lower levels of rainfall compared to the first year (Fig. 1). According to a study conducted by Korres et al. [15], it was observed that rising temperatures would primarily impact the spread of weeds, specifically those belonging to the C4 category. The increase in temperature would result in an expansion of their geographic range and facilitate the proliferation of invasive weed species like itchgrass, cogongrass, and witchweed. Additionally, the researchers discovered that in situations where there is a scarcity of water or nutrients, certain r-strategist weeds characterized by S–C-R traits, such as Palmer amaranth, large crabgrass, johnsongrass, and spurges, are more likely to dominate. In the research conducted by Cardoso et al. [28], it was observed that the weed population in the cotton field consisted of a total of 21 species. Among these species, monocotyledonous plants were found to have greater relative importance and dominance.

The higher total dry weight and economic yields observed in 2021 can be attributed to the increased precipitation and lower temperatures compared to the conditions experienced in 2022 (Fig. 1). These environmental factors played a crucial role in shaping favorable growing conditions and subsequently influencing the crop's productivity.

Overall, it is evident that longer irrigation intervals and the presence of weeds negatively impact all traits measured. Furthermore, the intercropping system can play a role in enhancing this trait, as demonstrated by the higher harvest index, 1000-seed weight, boll count, plant height, leaf greenness index, and cottonseed protein content observed in the cotton when intercropped with medicinal plants. The observed decline in total dry weight, economic yields, and LAI within the intercropping system can be primarily attributed to the significant reduction in the density of cotton, which has been reduced to 50%. According to the study conducted by Cardoso et al. [28], it was found that the absence of weed control led to a significant decrease of 82.9% in cotton yield.

According to a study by Yang et al. [29], in shade conditions caused by intercropping, there is an expected increase in plant height due to a reduction in the ratio of red to far red light (FR/R) and a decrease in the amount of photosynthetic active radiation. This increase in plant height can be attributed to competition for more light interception and limited light penetration through the canopy, as well as a lack of decomposition of auxin hormone under these intercropping conditions. Furthermore, a recent study by Amiriyan Chelan et al. [30] focused on intercropping *Dracocephalum moldavica* L. with fenugreek (*Trigonella foenum-graecum* L.). The researchers observed that the height of both species increased in the intercropping system compared to mono cropping.

Intercropping systems that focus on increasing cottonseed protein have demonstrated their potential to provide a higher nitrogen supply for plants. Consequently, the elevation in the aforementioned traits’ values can be attributed to the enhanced growth characteristics of plants resulting from increased nutrient absorption and accessibility and decreased soil-borne diseases. Zhang et al. [10] conducted a study that demonstrated a noteworthy reduction in the disease index of Cotton Verticillium wilt through intercropping with cotton-garlic and cotton-onion. Emerging evidence suggests that medicinal plants possess significant potential to influence the soil microbial community and enhance crop yield. Liu et al. [31] suggested that the availability of nitrogen (N) can play a significant role in controlling N metabolism, the growth and distribution of biomass, and ultimately influencing both the overall yield and specific components of seed cotton production.

On the other hand, decreased values observed in traits measured within weedy plots and longer irrigation intervals can be attributed to reduced water and nutrient availability. The findings of this study are consistent with the observations made by Baytar et al. [32], which indicated that water deficit stress has a negative impact on fiber quality, boll number, and plant height in various cotton genotypes. Under water deficit conditions, plants exhibit a diminished leaf net photosynthetic rate and carbon assimilation. This decline can be attributed to a decrease in the carbon concentration within the leaf and chloroplast damage. Consequently, inadequate water availability negatively impacts the plant's ability to carry out efficient photosynthesis and compromises its overall physiological processes [33].

Under water stress conditions, it was observed that the competitive ability and water use efficiency of cotton varieties increased. The occurrence and success of weeds in fields have been attributed to a general weediness syndrome characterized by their rapid resource capture [34]. Wang et al. [35] determined that the optimal volume of irrigation water for cotton growth is 675 mm. Moreover, their findings highlighted the potential consequences of excessive irrigation, specifically the movement of nitrogen into the deeper layers of the soil. Li et al. [36] reported that as the overall irrigation amount increased, there was a corresponding increase in cotton yield. However, simultaneously, the water use efficiency decreased.

The LER is a crucial intercropping evaluation index that quantifies the advantage of intercropping in optimizing land use. The analysis of LER values revealed that the weedy plots exhibited the highest values, indicating a greater efficiency in land utilization within those particular conditions. Previous research has consistently demonstrated that the superiority of intercropping can be attributed to various factors, including distinct morphological properties and growth patterns, as well as the inherent ability of intercropping components to efficiently utilize vital resources such as soil, moisture, light, and essential nutrient elements [30,37,38]. Wang et al. [38], reported that a jujube–cotton intercropping system had advantages in LER, yield, and income.

## 5 Conclusion

Overall, these findings highlight the significance of implementing weed control measures and adopting appropriate irrigation strategies to enhance cotton productivity. Intercropping emerges as a valuable approach in integrated weed management, particularly in scenarios with weed pressure. Moreover, the choice of cotton variety and irrigation interval can influence water use efficiency, and certain varieties may exhibit greater tolerance to environmental stresses such as moisture and weeds. The intercropping systems exhibited favorable LER indices, indicating higher productivity compared to sole cropping. Notably, the intercropping systems consistently demonstrated the highest LER indices under weedy conditions, highlighting the value of intercropping as an effective method for integrated weed management as recommended by Blaise et al. [39]. Furthermore, there were no significant differences in WUE among different cotton varieties. However, it was observed that increasing the irrigation interval in both years led to improved WUE. Conversely, the presence of weeds had a negative impact on WUE. Among the experimental factors examined, it was found that weeds exerted the most significant impact on seed quality. This was determined by evaluating protein and oil contents, which were observed to decrease in the presence of weeds. Specifically, the mono-cropped Golestan variety performed well under unstressed conditions, such as with an irrigation interval of 3 days and when grown in weed-free conditions. In contrast, the intercropped Hekmat variety displayed better resilience to both moisture and weed stresses.

## Funding information

This study was generously funded and supported by 10.13039/501100008257Tarbiat Modares University, Tehran, Iran.

## Ethics approval and consent to participate

Not applicable.

## Consent for publication

Not applicable.

## Data availability statement

The datasets used and/or analysed during the current study are available from the corresponding author on reasonable request.

## CRediT authorship contribution statement

**Basim Mohammed Abdulkareem:** Writing – review & editing, Writing – original draft, Project administration, Investigation, Data curation, Conceptualization. **Ali Mokhtassi-Bidgoli:** Writing – review & editing, Writing – original draft, Visualization, Validation, Supervision, Software, Resources, Project administration, Methodology, Investigation, Funding acquisition, Formal analysis, Data curation. **Mahdi Ayyari:** Writing – review & editing, Writing – original draft, Project administration, Data curation. **Eshagh Keshtkar:** Writing – review & editing, Writing – original draft, Project administration, Conceptualization. **Hamed Eyni-Nargeseh:** Writing – review & editing, Writing – original draft, Project administration, Conceptualization.

## Declaration of competing interest

The authors declare that they have no known competing financial interests or personal relationships that could have appeared to influence the work reported in this paper.
