# Supplementary material for: Enhancing cotton sustainability: Multi-factorial intercropping, irrigation, and weed effects on productivity, quality and physiology
Source: Heliyon. 2024 Feb 27;10(5):e27135. doi: 10.1016/j.heliyon.2024.e27135 (PMC10912634; doi:10.1016/j.heliyon.2024.e27135)
Supplement: Multimedia component 1 [file mmc1.docx]

a

c

b

Fig. S1 a) The three-way interactions of intercropping systems (four growth conditions including mono- and inter-cropped cotton varieties Golestan and Hekmat), irrigation (three intervals of 3, 6, and 9 days), and competition (weed-free and weedy plots) on the seed cotton harvest index (HI) of cotton in 2021 and the main effects of b) intercropping systems and c) competition in 2022. In each year, different letters denoted above the columns indicate statistically significant differences at a significance level of *P* ≤ 0.05.

Fig. S2 The three-way interactions of intercropping systems (four growth conditions including mono- and inter-cropped cotton varieties Golestan and Hekmat), irrigation (three intervals of 3, 6, and 9 days), and competition (weed-free and weedy plots) on the cottonseed harvest index (HI) of cotton in 2021 and 2022. In each year, different letters denoted above the columns indicate statistically significant differences at a significance level of *P* ≤ 0.05.

b

a

2021

2022

d

c

2022

2022

Fig. S3 The main effects of a and b) irrigation (three intervals of 3, 6, and 9 days), c) intercropping systems (four growth conditions including mono- and inter-cropped cotton varieties Golestan and Hekmat), and d) competition (weed-free and weedy plots) on the lint harvest index (HI) of cotton in 2021 and 2022. In each year, different letters denoted above the columns indicate statistically significant differences at a significance level of *P* ≤ 0.05.

a

b

Fig. S4 a) The main effect of competition in 2021 and b) the three-way interaction of intercropping systems (four growth conditions including mono- and inter-cropped cotton varieties Golestan and Hekmat), irrigation (three intervals of 3, 6, and 9 days), and competition (weed-free and weedy plots) on the 1000-seed weight of cotton in 2022. In each year, different letters denoted above the columns indicate statistically significant differences at a significance level of *P* ≤ 0.05.

b

a

c

d

Fig. S5 a) The main effects of irrigation (three intervals of 3, 6, and 9 days) and b) competition (weed-free and weedy plots) in 2021, and c) intercropping systems (four growth conditions including mono- and inter-cropped cotton varieties Golestan and Hekmat) in 2022, and d) the two-way interaction of irrigation and competition on the number of bolls per plant of cotton in 2022. In each year, different letters denoted above the columns indicate statistically significant differences at a significance level of *P* ≤ 0.05.

a

b

d

c

Fig. S6 a) The main effects of intercropping systems (four growth conditions including mono- and inter-cropped cotton varieties Golestan and Hekmat) in 2021, b) the two-way interaction of irrigation and competition in 2021, and the main effects of c) irrigation (three intervals of 3, 6, and 9 days) and d) competition (weed-free and weedy plots) in 2022 on the plant height of cotton. In each year, different letters denoted above the columns indicate statistically significant differences at a significance level of *P* ≤ 0.05.

a

b

c

Fig. S7 a) The two-way interaction of irrigation (three intervals of 3, 6, and 9 days) and competition (weed-free and weedy plots) in 2021 and the main effects of b) intercropping systems (four growth conditions including mono- and inter-cropped cotton varieties Golestan and Hekmat) and c) competition on the leaf greenness index of cotton in 2022. In each year, different letters denoted above the columns indicate statistically significant differences at a significance level of *P* ≤ 0.05.

a

b

Fig. S8 The biplots represent a principal components analysis of intercropping systems (four growth conditions including mono- and inter-cropped cotton varieties Golestan and Hekmat, first number of treatments 1, 2, 4, and 5), irrigation (three intervals of 3, 6, and 9 days, second number of treatments 1-3), and competition (weed-free and weedy plots, third number of treatments 1-2) on the total dry weight (Y1), cottonseed yield (Y2), lint yield (Y3), seed cotton yield (Y4), 1000-seed weight (Y5), Bolls no/plant (Y6), leaf greenness index (Y7), photosynthetic rate (Y8), plant height (Y9), LAI (Y10), cottonseed protein content (Y11), and cottonseed oil content (Y12) of cotton in a) 2021 and b) 2022.

a

c

b

e

d

Fig. S9 The main effects of a) intercropping systems (four growth conditions including mono- and inter-cropped cotton varieties Golestan and Hekmat), irrigation (three intervals of 3, 6, and 9 days) in b) 2021 and c) 2022, and competition (weed-free and weedy plots) on the water use efficiency (WUE) of cotton in d) 2021 and e) 2022. In each year, different letters denoted above the columns indicate statistically significant differences at a significance level of *P* ≤ 0.05.
